# High prevalence of fatty liver and its association with metabolic syndrome among rural adults with chronic hepatitis C: Implications for primary healthcare

**DOI:** 10.1186/s12889-024-17851-0

**Published:** 2024-02-20

**Authors:** Ta-Jen Wang, Mei-Yen Chen, Yu-Chih Lin, Wen-Nan Chiu, Tung-Jung Huang, Hsu-Huei Weng

**Affiliations:** 1 Department of Diagnostic Radiology, Chang Gung Memorial Hospital, Chiayi, Taiwan; 2 Department of Nursing, Chang Gung University of Science and Technology, Chiayi, Taiwan; 3 Department of Cardiology, Chang Gung Memorial Hospital, Chiayi, Taiwan; 4 Department of Family Medicine, Chang Gung Memorial Hospital, Chiayi, Taiwan; 5 Department of Internal Medicine, Chang Gung Memorial Hospital, Chiayi, Taiwan; 6 Department of Respiratory Care, Chang Gung University of Science and Technology, Chiayi, Taiwan; 7 School of Nursing, Chang Gung University, Taoyuan, Taiwan; 8 College of Medicine, Chang Gung University, Taoyuan, Taiwan

**Keywords:** Fatty liver, Chronic hepatitis C, Cardiometabolic diseases, Metabolic syndrome

## Abstract

**Background:**

Chronic hepatitis C (CHC) virus infection is a global health concern that is associated with significant liver-related morbidity and mortality. Owing to the inflammatory pathway, CHC can causefatty liver, liver cirrhosis, and liver cancer and is associated with cardiometabolic diseases, such as hypertension and diabetes. Fatty liver is associated with metabolic disorders, cardiovascular diseases, diabetes, and liver cancer. Hence, the early detection of fatty liver through noninvasive screening in adults with CHC is important in primary healthcare settings. This study aimed to explore the prevalence of fatty liver and its association with metabolic syndrome amongrural adults with CHC.

**Methods:**

This was a series of cohort studies related to the elimination of the CHC burden around the western coastal Yunlin County, Taiwan, between August 2018 and July 2021. A cross-sectional study was conducted after hepatitis C virus RNA confirmation in a hepatitis C- endemic area. A gastrointestinal physician or radiologist assessed fatty liver by ultrasonography. Fatty liver was classified into four grades: normal, mild, moderate, and severe. Three liver enzyme biomarkers were identified. According to the Taiwan national standard, metabolic syndrome was defined based on the presence of three or more of the five abnormal biomarkers, including increased waist circumference, elevated blood pressure, elevated fasting blood glucose level, elevated triglyceride level, and low high-density lipoprotein cholesterol level.

**Results:**

A total of 256 rural adults with CHC were enrolled. The mean age of the participants was 67.5 (standard deviation = 11.8) years, with a low educational level. High prevalence of fatty liver (79%), central obesity (54.3%), elevated blood pressure (55.5%),elevated fasting blood glucose (FBG) level (44.9%), and metabolic syndrome (37.9%) were observed.The results showed that adults with CHC with moderate to severe fatty liver were significantly associated with an increased risk of increased waist circumference (*P* < 0.001), increased blood pressure (*P* < 0.001), low high-density lipoprotein cholesterol level (*P* < 0.05), and elevated liver enzyme biomarker levels (all *P* < 0.05) after adjusting for age, sex, and educational level. Furthermore, adults with CHC with moderate to severe fatty liver were significantly associated with a greater risk of metabolic syndrome (odds ratio = 2.85, 95% confidence interval = 1.66 to 4.92).

**Conclusions:**

The findings demonstrate a high prevalence of fatty liver in rural adults with CHC, which is significantly associated with obesity, metabolic syndrome, and elevated liver biomarker levels. Clinicians and primary healthcare providers must encourage patients with CHC to receive antiviral therapy combined with weight loss management and lifestyle modification, allowing general improvements in their liver and cardiometabolic health.

## Background

Recently, the World Health Organization [1] estimated that globally, 58 million individualshad hepatitis C virus (HCV) infectionand approximately 290,000 people died from hepatitis C in 2019, mostly related to liver cirrhosis and hepatocellular carcinoma (HCC or liver cancer). HCC can be induced by viral hepatitis and obesity [1–3]. Chronic hepatitis C (CHC) is defined as an infection with HCV for more than 6 months andis a leading cause of liver-related morbidity and mortality worldwide. In Taiwan, the current prevalence rate of CHC is approximately 3–4% and is specifically prevalent in western costal southern Taiwan. HCC, an important comorbidity of CHC, is attributed to over 7000 deaths in Taiwan annually [2, 4]. Therefore, the eradication of HCV infection has become an urgent priority in the health policy of the Taiwanese government.

The pathophysiological mechanisms of CHC involve chronic inflammation leading to liver fibrosis, activation of pro-inflammatory cytokine mechanisms, and increased systemic oxidative stress causing systemic metabolic derangements and pro-oncogenic developments, such as oncogene activation and angiogenesis [5]. CHC is significantly associated with cardiometabolic diseases, such as hypertension, heart disease, and diabetes [6, 7]. Moreover, CHC has been associated with the development of hepatic steatosis and fatty liver via the impairment of hepatocyte metabolism and fat accumulation [8]. Fatty liver is defined as the presence of accumulation of intrahepatic fat of at least 5% of liver weight, which may occur from both HCV infection and metabolic derangements a result of obesity and canbe an early manifestation of insulin resistance, potentially leading to the development of type 2 diabetes [9–12].

Non-alcoholic fatty liver disease (NAFLD) is defined by fatty changes that occur in individuals without excessive alcohol consumption [9] and is an increasingly recognized public health issue affecting several adult populations worldwide [13]. Obesity is one of the most frequent causes of NAFLD and subsequent liver disease [3]. In several developed countries, NAFLD is associated with a moderately increased risk of cardiometabolic diseases, cardiovascular mortality, and liver cancer [14, 15]. Owing to the better understanding of liver pathophysiology and its association with metabolic abnormalities, metabolic dysfunction-associated fatty liver disease (MAFLD) has been proposed as a more appropriate term than NAFLD in the recent years [16–18].

Ultrasonography is the most commonly used imaging tool to establish the diagnosis of fatty liver because it is widely available, simple, well-established, reproducible, and inexpensive [15, 19]. Hence, early detection of fatty liver using ultrasonography for adults with CHC or obesity should be prioritized by primary healthcare providers, especially in rural areas [20, 21]. Therefore, this study aimed to explore the prevalence of fatty liver and its association with metabolic syndrome (MetS) in rural adults with CHC prior to receiving antiviral treatment.

## Methods

### Design, sample, and setting

**Fig. 1:**
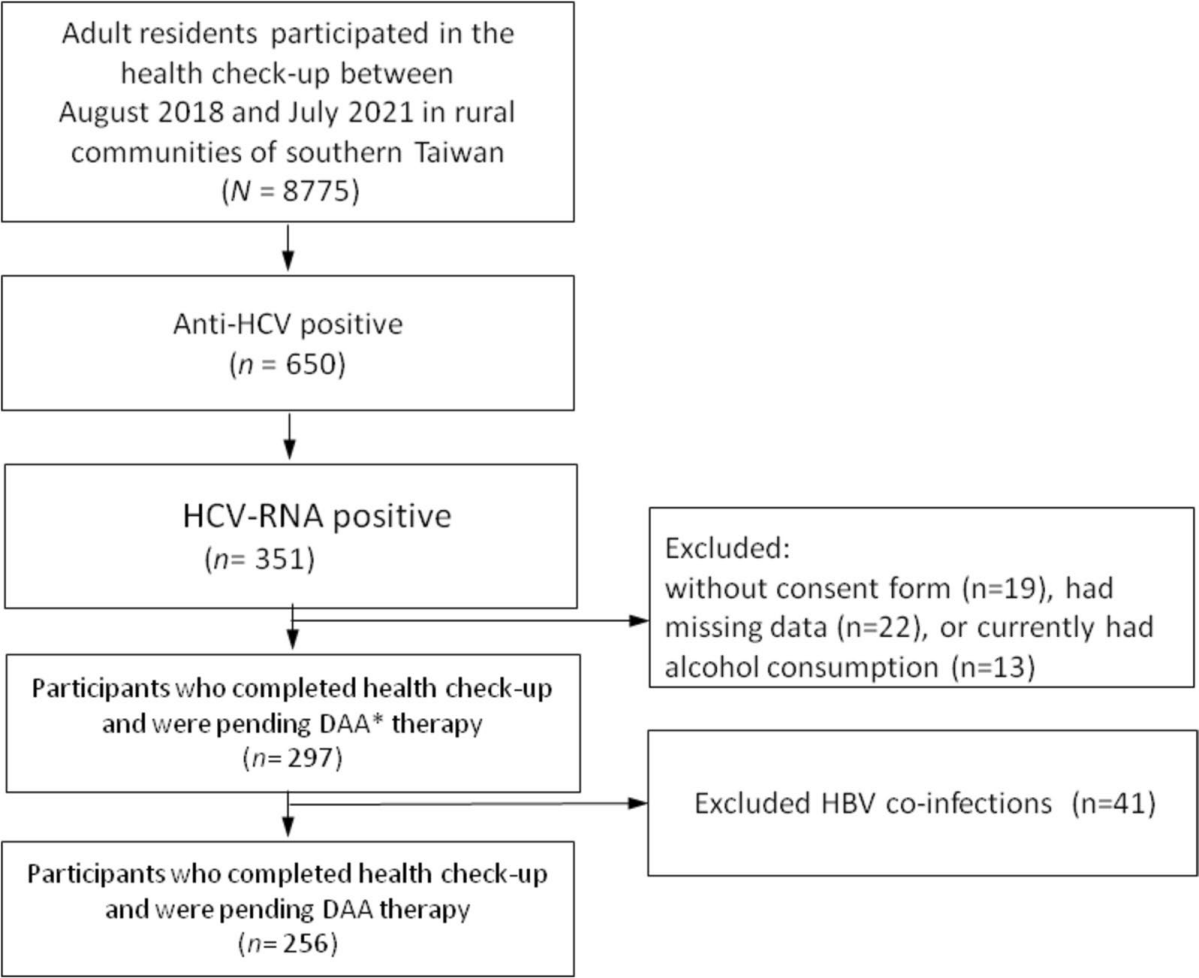
Flow chart of participants’ recruitment, inclusion and exclusion. Abbreviations: *DAA* Direct-acting antivirals

A cross-sectional study design with community-based health development was applied to this hepatitis C-endemic area. This was a series of cohort studies related to eliminating the CHC burden around the western coastal Yunlin County, Taiwan, between August 2018 and July 2021 [22]. During specific community liver health screening, rural adults who were anti-HCV-positive were invited to the collaborating hospital for further HCV RNA confirmation [23]. The participants were then referred to the collaborating hospital to receive antiviral therapy by the research team. The inclusion criteria were as follows: (1) fully independent in daily activity and able to walk to the local hospital, (2) age > 20 years and able to communicate in Mandarin or Taiwanese, and (3) agreed to participate in this study and signed the informed consent form. The exclusion criterion was as follows: (1) Currently had alcohol consumption. (2) Since hepatitis B infection may have an inverse effect on hepatic steatosis [24], patients co-infected with hepatitis B were excluded from the study. The participant selection flow chart was described in Fig. 1.

### Procedure and ethical considerations

Before conducting this study, the institutional review board (IRB) (IRB No. 201701919B0) was approved by the ethics committee of the Chang Gung Memorial Hospital. The research team described the study procedures, for example, withdrawal of blood samples after 8 h of overnight fasting to examine blood glucose and lipid levels in all the participants before they came to the collaborating local hospital. Face-to-face interviews were conducted at outpatient clinics, and informed consent was obtained from all literate participants, and from legally acceptable representatives for a small portion of participants who were illiterate. The research assistant measured the participants’ body weight, body height, waist circumference (WC), and blood pressureon the same day. Physiological biomarkers were recorded in the medical charts of the collaborating hospital.

#### 1. Demographic measurements

Demographic characteristics included sex, age, education level (years of education), body weight, body height, and body mass index (BMI, body weight in kilogram/body height in m^2^). The participants were asked whether they smoked cigarettes, chewed betel nuts, and consumed alcohol (never, quitted, and currently drinking. Currently drinking was defined as 20 g alcohol daily, which is approximately equivalent to 400 mL of 5% beer).

#### 2. Metabolic syndrome and liver biomarkers

MetS was defined by the Taiwan national standard [2], which included the presence of three or more of the following cardiometabolic risk factors: (a) central obesity: WC ≥ 90 and 80 cm in men and women, respectively (WC was measured between the last rib margin and iliac crest (the mid-abdominal distance); (b) systolic blood pressure (SBP)/ diastolic blood pressure (DBP)> 130 and 85 mmHg, respectively; (c) serum high-density lipoprotein cholesterol (HDL-C) levels< 40 and 50 mg/dL in men and women, respectively; (d) serum FBG level >100 mg/dL; and (e) serum triglyceride (TG) level > 150 mg/dL. Any participant currently using medications for hypertension, diabetes mellitus, or hyperlipidemia was classified as having elevated cardiometabolic risk. In addition, three liver biomarkers, serum glutamic-oxaloacetic transaminase (GOT), glutamic-pyruvic transaminase (GPT), and gamma-glutamyl transferase (GGT), were collected in this study.

#### 3. Ultrasonographic screening of fatty liver disease

Ultrasound examinations in the supine position were performed using three units with curved array transducers. Ultrasonography assessment was performed by a team comprised of 3 gastrointestinal physicians and 2 radiologists, all with 6 to 10 years of working experience in abdominal ultrasonography. The diagnosis of fatty liver was established based on the brightness of the liver compared to the kidney, vascular blurring of the hepatic vein trunk, and deep attenuation in the right hepatic lobe [25]. The severity of fatty liver change was divided into four grades (0–3): Grade 0, normal liver, normal echo texture, and no fatty change; Grade 1, mild fatty liver and mild increase in fine echoes in the parenchyma with slightly impaired visualization of the intrahepatic vessels and diaphragm; Grade 2, medium-grade diffuse increase in hepatic echogenicity and mild deterioration in the image of the diaphragm and intrahepatic vessels; Grade 3, moderate to severe fatty liver and marked increase in fine echoes in the parenchyma with poor or no visualization of the intrahepatic vessel borders, diaphragm, and posterior right lobe of the liver.

### Data analysis

Based on the grades of abdominal sonography, the participants were classified into two groups: no/mild (grades 0–1) and moderate to severe (grades 2–3) fatty liver. The demographics and characteristics of the participants between groups were compared using an independent sample t-test for continuous variables, the Mann–Whitney U-test for apparently skewed data (e.g., liver biomarkers), and the chi-squared test for categorical variables. The association between moderate to severe fatty liver and cardiometabolic risk factors (e.g., WC) was assessed using linear regression analysis. The association between moderate to severe fatty liver and liver biomarkers was evaluated using quantile regression, in which the 50th percentile (median) was set as the dependent variable. Finally, the association between moderate to severe fatty liver and the risk of abnormality for cardiometabolic risks (e.g., central obesity) and MetS was investigated using logistic regression analysis. Several covariates, including age, sex, and educational level, were adjusted for in the regression models. All tests were two-tailed, and p < 0.05 was considered statistically significant. Data analyses were performed using the Statistical Package for the Social Sciences version 26 (IBM SPSS Inc., Chicago, Illinois, USA).

## Results

### Characteristics of the participants

A total of 256 rural adults with CHC were conveniently included for analysis, of whom female was more predominant than male (*n* = 169, 66%). The mean age of the participants was 67.5 (standard deviation [SD] = 11.8) years. The average educational level was relatively low, with a mean of 4.3 (SD = 5.0)years. Regarding the abnormalities of MetS components, the prevalence was highest for elevated blood pressure (55.5%) and central obesity (54.3%), followed by elevated FBG level (44.9%), and low HDL-C level (43%) and was lowest for TG level (13.7%). Ninety-seven (37.9%) participants had MetS, and the average number of MetS components was 2.1 (SD = 1.3) (Table 1).

**Table 1:** Demographics characteristics of the chronic hepatitis C adults with or without moderate to severe fatty liver

Variables	Total (*n* = 256)	Moderate/Severe (*n* = 103)	None/Mild (*n* = 153)	*P* value
Demographics				
Female	169 (66.0)	72 (69.9)	97 (63.4)	0.346
Age, year	67.5 ± 11.8	67.7 ± 10.8	67.4 ± 12.5	0.829
Education level, year	4.3 ± 5.0	4.0 ± 4.8	4.5 ± 5.2	0.481
Body mass index, kg/m^2^	25.6 ± 3.6	26.7 ± 3.6	24.9 ± 3.4	< 0.001
Substance use				
Smoking	59 (23.2)	26 (25.2)	33 (21.9)	0.548
Betel nut chewing	16 (6.3)	6 (5.8)	10 (6.6)	1.000
Components of MetS				
Waist circumference (WC), cm	84.1 ± 9.2	86.8 ± 8.8	82.2 ± 9.0	< 0.001
Systolic blood pressure, mmHg	132.0 ± 17.8	134.6 ± 17.2	130.3 ± 18.0	0.061
Diastolic blood pressure, mmHg	75.2 ± 11.3	77.2 ± 12.3	73.8 ± 10.4	0.019
Fasting blood glucose, mg/dL	105.1 ± 24.8	107.4 ± 28.3	103.6 ± 22.0	0.221
Triglyceride, mg/dL	100.1 ± 48.6	107.2 ± 53.3	95.3 ± 44.7	0.055
High-density lipoprotein, mg/dL	50.6 ± 14.4	48.5 ± 14.3	52.0 ± 14.2	0.056
Abnormal components of MetS				
Elevated central obesity (WC)^a^	139 (54.3)	71 (68.9)	68 (44.4)	< 0.001
Elevated blood pressure^b^	142 (55.5)	68 (66.0)	74 (48.4)	0.007
Elevated fasting blood glucose^c^	115 (44.9)	53 (51.5)	62 (40.5)	0.096
Elevated triglyceride^d^	35 (13.7)	17 (16.5)	18 (11.8)	0.354
Abnormal HDL-C^e^	110 (43.0)	54 (52.4)	56 (36.6)	0.014
Metabolic syndrome (MetS)^f^	97 (37.9)	54 (52.4)	43 (28.1)	< 0.001
Number of components of MetS	2.1 ± 1.3	2.6 ± 1.3	1.8 ± 1.2	< 0.001
Liver function				
GOT, U/L	31.5 [24.0, 51.0]	33.0 [24.0, 57.0]	29.0 [24.0, 43.0]	0.030
GPT, U/L	33.5 [23.0, 55.5]	37.0 [27.0, 74.0]	31.0 [22.0, 47.0]	0.022
GGT, U/L	26.5 [17.0, 51.5]	31.0 [19.0, 66.0]	24.0 [16.0, 43.0]	0.002

*Abbreviation*: *GOT* Glutamic-oxalocetic transaminase, *GPT* Glutamic-pyruvic transaminase, *GGT* Gamma-glutamyl transferase

Data were presented as frequency (percentage), mean ± standard deviation or median [25th percentile, 75th percentile];

^a^WC, waist circumference, male > 90 cm, female > 80 cm;

^b^Blood pressure > 130/85 mmHg; systolic blood pressure / diastolic blood pressure;

^c^Fasting blood glucose ≥ 100 mg/dL;

^d^Triglyceride ≥ 150 mg/dL;

^e^HDL, high-density lipoprotein cholesterol, male < 40 mg/dL, female < 50 mg/dL;

^f^Number of components of MetS ≥ 3

**Fig. 2:**
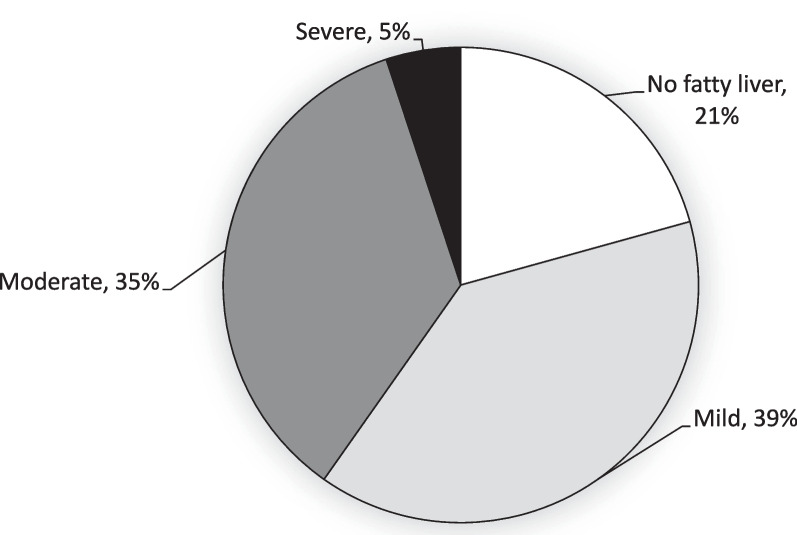
The distribution of fatty liver severity among rural adults with chronic hepatitis C

Among the 256 participants, 100 (39%) had mild, 90 (35%) moderate, and 13 (5%) severe fatty liver (Fig. 2). Compared with participants with no or with mild fatty liver, those with moderate to severe fatty liver had higher average BMI (*P* < 0.001), WC (*P* < 0.001), SBP(*P* < 0.05), DBP (*P* < 0.001), TG level (*P* < 0.05), and lower HDL-C level (*P* < 0.05). Those with moderate or severe fatty liver also had more abnormal MetS components and had anoverall higher prevalence of MetS than those with no or mild fatty liver (*P* < 0.001). In addition, the liver biomarker levels were significantly elevated in the moderate to severefatty liver group, including GOT (*P* < 0.05), GPT (*P* < 0.05), and GGT (*P* < 0.001) (Table 1).

### Association among moderate to severe fatty liver, liver biomarkers, and metabolic syndrome

Table 2 shows that participants with moderate to severe fatty liver were significantly associated with elevated GGT levels (regression coefficient [*B*] = 7.92, 95% confidence interval [CI] = 0.48 to 15.36). The moderate to severe grade of fatty liver was borderline significantly associated with greater GOT levels (*B* = 5.54, 95% CI = -0.19 to 11.27, *P* = 0.058) Participants with moderate to severe fatty liver were significantly associated with a higher mean difference of most components of MetS, with systolic blood pressure and fasting blood glucose level being the exceptions. Table 3 demonstrates that moderate to severe fatty liver was significantly associated with larger WC (odds ratio [OR] = 2.71, 95% CI = 1.59to 4.64), elevated blood pressure (OR = 2.08, 95% CI = 1.21 to 3.58), and lower HDL-C level (OR = 1.94, 95% CI = 1.16to 3.24). Overall, moderate to severe fatty liver was significantly associated with the diagnosis of MetS (OR = 2.86, 95% CI = 1.66 to 4.92).

**Table 2:** The association between moderate to severe fatty liver, liver function and components of metabolic syndrome

Outcome	Regression coefficient (95% CI)^a^	*P* value
Liver function^b^		
GOT, U/L	5.54 (-0.19 to 11.27)	0.058
GPT, U/L	3.63 (-2.93 to 10.19)	0.277
GGT, U/L	7.92 (0.48 to 15.36)	0.037
Components of MetS^**c**^		
Waist circumference, cm	4.93 (2.85 to 7.02)	< 0.001
Systolic blood pressure, mmHg	3.72 (-0.49 to 7.94)	0.084
Diastolic blood pressure, mmHg	3.26 (0.48 to 6.03)	0.021
Fasting blood glucose, mg/dL	3.51 (-2.46 to 9.48)	0.249
Triglyceride, mg/dL	12.41 (0.39 to 24.42)	0.043
High-density lipoprotein, mg/dL	-4.14 (-7.40 to -0.87)	0.013
Number of components of MetS	0.70 (0.41 to 0.99)	< 0.001

*Abbreviation*: *CI* Confidence interval, *GOT* Glutamic-oxalocetic transaminase, *GPT* Glutamic-pyruvic transaminase, *GGT* Gamma-glutamyl transferase. *MetS* Metabolic syndrome

^a^Adjusted for age, sex and education level

^b^Quantile regression

^c^Linear regression

**Table 3:** The association between moderate to severe grade of fatty liver and the metabolic syndrome

Outcome	Odds ratio (95% CI)^g^	*P* value
Elevated WC^a^	2.71 (1.59 to 4.64)	< 0.001
Elevated SBP/DBP^b^	2.08 (1.21 to 3.58)	0.008
Elevated FBG^c^	1.52 (0.90 to 2.56)	0.118
Elevated TG^d^	1.57 (0.76 to 3.24)	0.222
Low HDL-C^e^	1.94 (1.16 to 3.24)	0.012
Metabolic syndrome (MetS)^f^	2.86 (1.66 to 4.92)	< 0.001

*Abbreviation*: *CI* Confidence interval

^a^*WC* Waist circumference, male > 90 cm, female > 80 cm

^b^Blood pressure > 130/85 mmHg; systolic blood pressure /diastolic blood pressure

^c^Fasting blood glucose ≥ 100 mg/dL

^d^Triglyceride ≥ 150 mg/dL

^e^HDL, high-density lipoprotein cholesterol, male < 40 mg/dL, female < 50 mg/dL

^f^Number of components of MetS ≥ 3

^g^Adjusted for age, sex and education level

## Discussion

In Taiwan, few studies have focused on the prevalence of fatty liver and its association with MetS among rural adults with CHC. Two important findings are revealed in this study. First, a high prevalence of rural adults with CHC had fatty liver and cardiometabolic risk factors. Second, rural adults with CHC who had moderate to severe fatty liver were significantly associated with a greater risk of MetS and elevated liver biomarker levels.

Our study demonstrated that more than three-quarters of the rural adults with CHC presented with fatty liver, and 39% of all patients had mild steatosis, while 40% had moderate to severe steatosis. The prevalence of fatty liver is consistent with the finding of a review study by Siphepho et al. [12], who reported that most patients with CHC had varying degrees of steatosis within the periportal region of the liver, having an estimated prevalence of 40%–86%, although they reported that 78% of patients presented with mild steatosis. A study conducted in Germany by Rau et al. [26] found that only 12.3% of patients with CHC presented with fatty liver. The possible reasons to differences in severity of fatty liver might be due to the difference in HCV genotypes leading to differing disease progression or physician’s inter-rater reliability of subjective diagnosis by ultrasound between different studies. As reported in the literature, patients with HCV genotype 3 show a higher degree of hepatic steatosis [26]. However, the major genotypes of HCV in Taiwan are types 1b and 2 [27]. Further studies are required to explore the association between genotype and fatty liver and related cell damage.

Despite the difference in fatty liver among CHC in different countries, our results showed significantly higher prevalence of fatty liver than the general adult population in Asia with 29.6% [28], and also higher than that in Europe and the United States, with the prevalence of fatty liver being 25–30% [10]. Moreover, our study revealed that more than half of the participants had central obesity and that more than one-third of the participants had MetS. Participants of our study are also found to be predominantly elderly, and had only 4.5 average years of education received. As the cause of fatty liver also included obesity, which has been associated with an unhealthy lifestyle, it is possible that our patients with CHC might have been affected by lower socioeconomic status, have lower health literacy in weight loss management (e.g., low levels of physical activity and excess calorie intake relative to expenditure in nutritionally imbalanced and unhealthy diets) [3, 16], and thereforehave a tendency to adopt less healthy lifestyles. A study in the United States, Giammarino et al. [29] demonstrated an association between socioeconomic deprivation of patients and the development of steatohepatitis and severe hepatic steatosis. Therefore, further studies are necessary to explore the association between socioeconomic factors and the development of fatty liver in rural adults with CHC.

Previous studies have also associated CHC with other cardiometabolic diseases, such as MetS and diabetes mellitus [11, 12, 30]. Hsiao et al. [30] reported that 62.1% of participants with CHC had MetS, 48.4% had hypertension, and 37.9% had diabetes. Recently, several researchers have opted the newly coined term “MAFLD,” replacing the previous NAFLD term. The redefinition reflects substantial information gained from previous studies on the association between fatty liver and overweight/obesity, type 2 diabetes, and metabolic risk factors [31–33]. We also like to use the term MAFLD, instead of NAFLD or fatty liver, as this term more accurately emphasizes cardiovascular risks in patients with CHC [34].

Considering the new era of CHC therapy via Direct-Acting antivirals(DAAs), DAAs are associated with > 95% cure rate and reduction in risk of developing HCC and are significantly beneficial in reducing diabetes mellitus, the levels of liver enzymes (GOT, GPT, and GGT) [33, 35], and the severity of fatty liver disease[36]. Hence, eradication of CHC through DAAs therapy may prevent adverse outcomes of cirrhosis and HCC and is potentially beneficial for the regression of fatty liver and control of diabetes mellitus. CHC treatment should be integrated with lifestyle management for patients with CHC to treat the disease and control the progression of hepatic steatosis and other cardiometabolic disorders.

### Limitations

Although this study revealed some valuable findings, it has several limitations. First, our sampling was not random, and this study was conducted in only one county. This may limit the representativeness and generalizability of the study’s findings. Second, we did not collect certain aspects of each participant’s history of cardiometabolic diseases, such as hypertension and diabetes, or medication history relevant to these diseases. This might lead to an underestimation of the prevalence of cardiometabolic risk factors or MetS and their contribution to steatosis. Third, the sample size of this study is relatively small with a relatively high number of variables, it is challenging to analyze possible interaction effects and rule out all confounding factors. Future large-scale studies are warranted to confirm the findings of this study. Fourth, our questions to participants regarding alcohol consumption did not precisely reflect different concentrations of alcohol in alcoholic beverages, and we did not account for the degree of past alcohol consumption for those who previously had regular alcohol consumption but had quitted. This may lead to underestimation of prevalence of alcohol-associated hepatic steatosis and other alcoholic related metabolic abnormalities, as well as the long-term effect of past alcoholism on the current hepatic metabolic conditions of some participants. Finally, health-related lifestyle factors were not explored in this study, limiting the available recommendations for this population.

## Conclusions

These findings demonstrate a high prevalence of fatty liver and cardiometabolic risk factors in adults with CHC living in rural regions. Adults with CHC with moderate and severe fatty liverare significantly associated with central obesity, elevated blood pressure, MetS, and elevated liver enzyme levels. This study highlights that clinicians and primary healthcare providers should encourage patients with CHC to undergo fatty liver screening in addition to DAAs treatment and to improve their general liver and cardiometabolic health through weight loss management and lifestyle modifications.

## Data Availability

The datasets analyzed during the current study are available from the corresponding author on reasonable request.

## Abbreviations

CHC: Chronic hepatitis C
DAA: Direct-acting antivirals
FBG: Fasting blood glucose
GGT: Gamma-glutamyl-transferase
GOT: Glutamic-oxalocetic transaminase
GPT: Glutamic-pyruvic transaminase
HCV: Hepatitis C virus
HDL-C: High-density lipoprotein cholesterol
MAFLD: Metabolic dysfunction-associated fatty liver disease
MetS: Metabolic syndrome
NAFLD: Non-alcoholic fatty liver disease
TG: Triglyceride
